# Non-canonical subunit functions in multiprotein complexes: insights from the Mediator complex

**DOI:** 10.1042/BST20260729

**Published:** 2026-06-22

**Authors:** Alexis Verger, Didier Monté, Vincent Villeret

**Affiliations:** CNRS UMR 9031 Biologie Structurale Intégrative, Univ. Lille, Institut Pasteur de Lille, F-59000 Lille, France

**Keywords:** independent subunit function, Mediator complex, multiprotein complex

## Abstract

The Mediator complex is a central regulator of RNA polymerase II transcription, integrating signals from transcription factors and coordinating pre-initiation complex assembly. Beyond this canonical role, numerous studies have implicated Mediator complex in diverse cellular processes, including RNA processing, DNA repair, and translational control. These observations raise a fundamental question: do such functions reflect an expansion of Mediator activity as a complex, or the emergence of specialized functions at the level of individual subunits? In this review, we examine the ability of Mediator subunits to function independently of their canonical context and discuss the mechanistic principles that enable subunit specialization within multiprotein assemblies.

## Introduction

**Figure 1:**
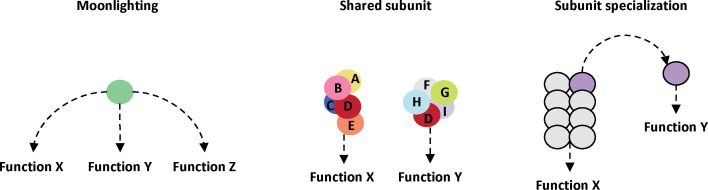
Different functional states as drivers of protein function From left to right: Moonlighting or multitasking proteins perform two or more independent and unrelated functions. Two distinct multiprotein complexes (subunits ABCDE and DFGHI) with different functions share a common subunit (subunit D). In a subunit specialization scenario, a subunit dissociates from the complex and carries out an additional function.

Multiprotein complexes govern most fundamental cellular processes, from transcription and translation to chromatin organization, signal transduction, and protein degradation. Within such assemblies, individual subunits are generally assumed to function as integral parts of the assembled machinery whose functions are inseparable from the intact architecture [1,2]. The Mediator complex exemplifies this paradigm. As a conserved, multisubunit co-regulator of RNA polymerase II (RNA Pol II) transcription, Mediator integrates signals from transcription factors and coordinates pre-initiation complex assembly at gene promoters and enhancers [3–6]. However, an increasing number of studies have attributed additional functions to Mediator in processes that extend beyond classical transcriptional regulation, including RNA processing and DNA repair [7–10]. These observations raise an important conceptual question: do they reflect previously unrecognized functions of Mediator as a complex, or do they instead arise from specialized activities of individual subunits? A key difficulty in addressing this question is that perturbation of Mediator subunits often results in widespread transcriptional changes, making it challenging to distinguish direct, transcription-independent functions from indirect effects on gene expression [11–13]. This ambiguity is not unique to Mediator but reflects a broader issue in the study of multiprotein assemblies [14]. Moreover, some subunits are shared between distinct multiprotein complexes or moonlight in alternative contexts [15,16], further complicating functional attribution (Figure 1). In moonlighting proteins, a single polypeptide performs multiple independent biochemical functions without reliance on complex architecture [17]. For example, an increasing number of key cytoplasmic metabolic enzymes are also known to directly regulate chromatin and transcription in the nucleus [18,19]. In contrast, shared subunits function as stable components of multiple, structurally defined complexes [20]. Recent work on the Mediator complex provides a concrete illustration of this conundrum. The Mediator subunit MED19, whose primary identity is defined by its incorporation into the complex, can localize to the nucleolus and regulate rRNA 2′-O-methylation [21]. MED19 provides one of the clearest indications that a Mediator subunit may acquire functions outside the canonical complex. However, rigorous demonstration of genuine subunit autonomy remains limited and represents an important challenge for future studies. Here, we review evidence supporting Mediator subunit independent functions and discuss their mechanistic basis and potential functional consequences within multiprotein complexes.

## Structural and assembly constraints

**Figure 2:**
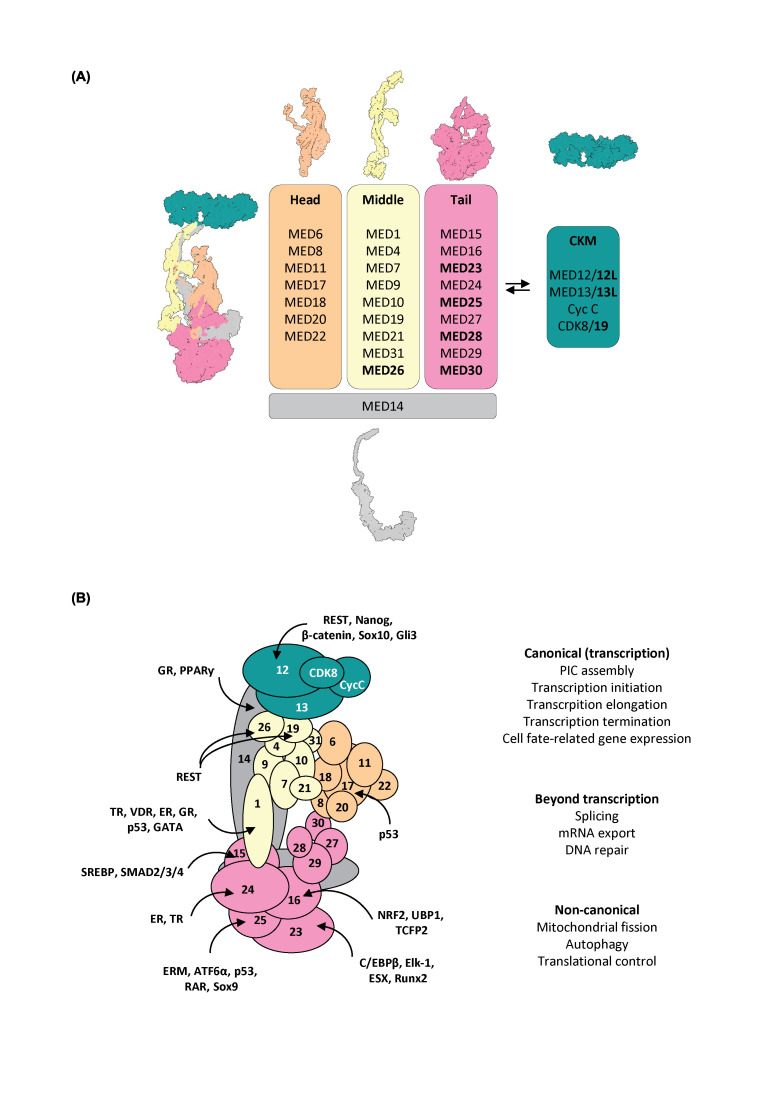
Subunit composition of human Mediator complex and interactions with transcription factors (**A**) Atomic model of human MED–CKM (PDB 8TQW) in surface representation. Mediator comprises four distinct modules: Head (in orange), Middle (in yellow), Tail (in pink), and the cyclin-dependent kinase 8 (CDK8) kinase module (in green), which is transiently associated with the complex. MED14, which links all three main modules (Head, Middle, and Tail), is indicated in grey. Mediator comprises 30 subunits in humans. Subunits with no yeast homologs are in bold. Adapted from [61]. (**B**) Several representative transcription factor–Mediator subunit interactions in mammals are indicated, adapted from [46,81]. The many functions of Mediator discussed throughout the text are indicated.

Mediator is a large, modular complex composed of Head, Middle, and Tail modules, together with a reversibly associated kinase module (Figure 2). Mediator facilitates assembly of the pre-initiation complex (PIC) by bridging transcription factors at enhancers with the basal transcription machinery at promoters [3]. Beyond its function in transcription initiation, the Mediator complex coordinates the processes of transcription elongation [22] and termination [23]. Two decades of structural studies have revealed a clear architectural hierarchy [24–29]. Subunits such as MED14 and MED17 form central scaffolds essential for complex stability [30–33]. Disruption of these architectural subunits typically destabilizes Mediator globally, making it difficult to attribute resulting phenotypes to potential independent subunit activity [11,12,34]. To address these limitations, recent studies have used rapid and reversible subunit depletion to investigate Mediator’s global functions in gene regulation and genome organization [11,12,35–40]. However, this systems-level approach may overlook the diverse, potentially non-canonical functions of individual subunits that now merit closer examination.

The structural and assembly constraints are further reinforced by co-translational assembly mechanisms, whereby subunits engage interaction partners during synthesis, limiting the accumulation of free, functional pools [41]. In such cases, individual subunits may be unstable or non-functional outside the assembled complex, as proper folding and solubility depend on interaction interfaces formed during assembly [24,42]. In contrast, more peripheral subunits, such as MED1, MED23, or MED25, occupy exposed positions (Figure 2) and engage dynamically with transcription factors [25–27,43–46]. Returning to MED19, this subunit is located at the very top of the complex (Figure 2), and previous data from yeast indicate that, under mild conditions, the majority of the ΔMED19 (Rox3) Mediator complex remains largely intact [47]. Such characteristics may support the existence of free subunit pools and create the potential for Mediator-independent function. Together, these observations suggest that subunit independence is not expected to be a general property, but rather a constrained and selective phenomenon.

## Emerging evidence for possible subunit-independent functions

**Figure 3:**
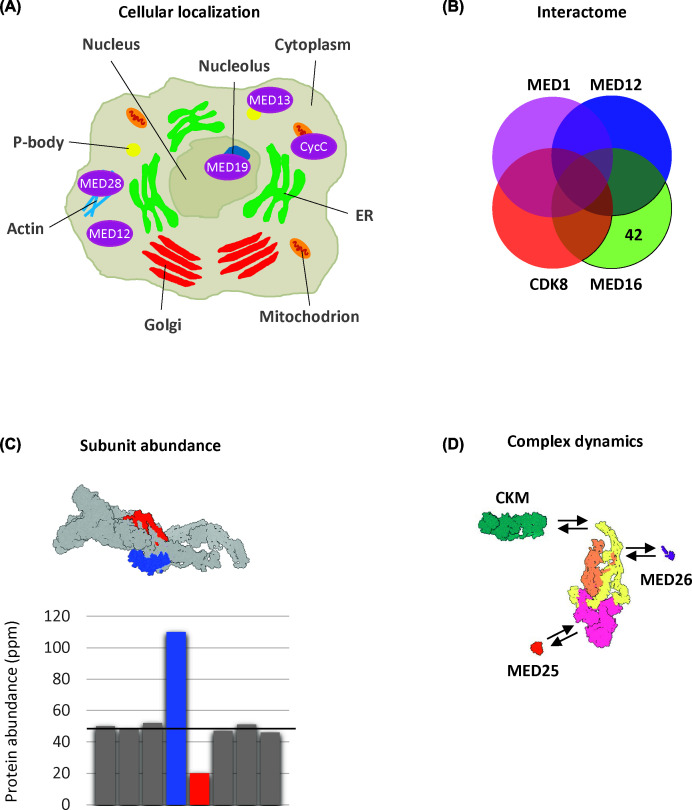
Functional specialization of subunits within multiprotein assemblies (**A**) Subcellular localization as a driver of Mediator subunit function. (**B**) Differential interactome. Venn diagram of MED1, MED12, CDK8, and MED16 interacting proteins identified by IP-MS, adapted from [23,91]. MED16 specific interacting proteins are highlighted. (**C**) Subunit abundance. The structure of Mediator complex is shown above the bar-plots of the cellular abundances of its respective subunits. The subunits that are expressed the most above (MED21) or below (MED25) the level expected based on their stoichiometry in the complex are highlighted in blue and red, respectively. Adapted from [97,98]. Protein abundance (ppm). (**D**) Compositional dynamics of Mediator. Dynamic association of subunits (CKM, MED26, and MED25) with core Mediator.

A subset of Mediator subunit functions occurs in contexts that are spatially or mechanistically incompatible with transcription. In this view, cellular localization may act as a gating mechanism that enables specialization without disrupting canonical Mediator function (Figure 3A) [48]. Among currently available examples, MED19, which serves as the cornerstone of the ideas explored in this review, provides robust evidence that a Mediator subunit may acquire functions outside the canonical complex. Although canonically embedded with the Middle module (Figure 2), human MED19 was recently shown to localize to the nucleolus (Figure 3A) [21]. It binds ribosomal RNA and fibrillarin (FBL) through a poly-lysine motif, promoting rRNA 2′-O methylation and enhancing internal ribosome entry site-mediated translation of select mRNAs. The high conservation of this lysine-rich C-terminal sequence across metazoans [21] underscores a possible evolutionary preservation of this independent function and its biological relevance. The emergence of an alternate MED19 isoform without the lysine-rich C-terminal sequence [49] also suggests that this conserved domain may be dispensable in certain contexts. In any case, this MED19 ribosomal function is mechanistically and spatially distinct from Mediator-dependent transcriptional regulation and does not appear to require intact complex assembly [21]. Although direct biochemical demonstration of endogenous free MED19 pools remains lacking, this example suggests that at least some Mediator subunits may acquire mechanistically separable activities outside the intact complex.

The metazoan-specific subunit MED28 (also known as EG-1 (endothelial-derived gene-1) or Magicin (merlin and Grb2 interacting cytoskeletal protein)) was originally identified as a cytoplasmic protein associated with actin-rich structures [50]. Although MED28 acts canonically in gene regulatory program to suppress smooth muscle differentiation [51], its involvement in cytoskeletal organization, cell migration, and Src-dependent signaling pathways [50,52] suggests that MED28 may shuttle between the nucleus and the cytoplasm through mechanisms that have yet to be identified. Notably, the majority of Mediator subunits lacks a recognizable nuclear localization signal, suggesting that they may use alternative nuclear import pathways such as importin-α-dependent piggybacking mechanism [53]. Similarly, MED15 has been shown to promote epithelial-mesenchymal transition and metastasis in bladder cancer by binding and stabilizing YAP1 through inhibition of ubiquitination. However, direct evidence for Mediator-independent function is still lacking, leaving open whether MED15 operates as a free subunit [54].

## Specific case: the kinase module

While the architectural hierarchy of Mediator generally restricts subunit independence, the kinase module (CKM) provides a striking exception. The CKM is conserved from yeast to humans and is composed of four subunits: the CDK8 kinase, Cyclin C, MED12, and MED13. In vertebrates, three additional paralogs, CDK19, MED12L, and MED13L, can also associate with Cyclin C in a mutually exclusive manner [55,56]. Unlike the three core Mediator modules (Head, Middle, and Tail), the kinase module is not constitutively associated with the complex and can reversibly bind or dissociate. Many studies have documented this reversible binding of CKM to Mediator [57–60], but the mechanistic details were elusive until recently [61]. A large intrinsically disordered region in MED13 (MED13 IDR) that is involved in core Mediator binding, blocks MED26 and RNA Pol II carboxy-terminal domain interaction to repress Mediator and regulates the transition from CKM–MED to MED–PIC [62,63]. This intrinsic modularity allows CKM subunits to function both within and outside Mediator. In particular, CKM subunits in yeast have been implicated in mitochondrial dynamics and autophagy [64], functions that are not easily reconciled with transcriptional control. In the cytoplasm, yeast Cyclin C is required for stress-induced mitochondrial hyperfission and promotes regulated cell death pathways [65,66]. Intriguingly, MED13 appears to act as a physical tether, keeping Cyclin C in the nucleus [67], a particularity also conserved in mammals [68,69]. Yeast MED13 is also able to translocate to cytoplasmic processing bodies under nitrogen starvation where it is required for efficient recruitment and autophagic degradation of the decapping protein Edc3 [70]. A further illustration of localization-linked subunit specialization is provided by mammalian MED12 (Figure 3A). Loss of MED12 confers chemoresistance to a range of cancer drugs in many cancer cell lines [71,72]. In all these cases, the impact of MED12 was shown to be independent of the Mediator complex. Instead, cytoplasmic MED12 interacts with the immature form of transforming growth factor beta receptor 2 (TGF-βR2) and inhibits its glycosylation, thereby preventing cell-surface expression. Consequently, downregulation of MED12 enhances TGF-βR2 cell-surface expression post-transcriptionally and activates TGFβ signaling [71,72]. The CKM module demonstrates how modular dissociation enables context-dependent specialization, whereas MED19 illustrates the potential for complete functional repurposing. These contrasting examples underscore the versatility of Mediator subunits in acquiring specialized roles through distinct mechanisms.

## How can subunit specialization occur?

Beyond transcription, Mediator has been implicated in a wide range of biological processes (Figure 2B). Within the nucleus, Mediator subunits participate in transcription-coupled RNA processing, including alternative splicing [73] and mRNA export [74]. Mediator has also been linked to chromatin organization [75], DNA repair [9], and cellular signaling pathways [76–80]. MED31 exemplifies an expanded Mediator-associated role in yeast by linking Mediator to the nuclear pore-associated TREX-2 complex to coordinate transcription with mRNA export [74]. Again, the key question is whether the Mediator subunits involved act within the Mediator complex, independently, or as components of a distinct, more specialized complex.

A substantial fraction of Mediator subunit-specific functions can be explained within the framework of transcriptional regulation, yet still display a high degree of biological specificity (Figure 2B). In these cases, individual subunits contribute to discrete gene expression programs in a manner that depends on cellular context, developmental stage, or interaction with specific transcription factors [6,46,81]. For example, MED1 plays a key role in erythroid differentiation through interaction with GATA-1 [82], illustrating how selective recruitment confers lineage specificity. Similarly, functions of MED1, MED14, and MED23 have been implicated in adipocyte differentiation [79,83–87], reflecting combinatorial control of transcriptional programs. Additional roles for MED12 [88] and MED13 [89] in lineage-specific enhancer regulation, as well as contributions of MED19 and MED26 to neuronal differentiation [90], further illustrate this principle. In all of these cases, functional specificity arises from selective recruitment, combinatorial assembly, and context-dependent transcriptional control, rather than independence from the Mediator complex.

Despite known biases of immunoprecipitation-mass spectrometry (IP-MS) experiments such as differences in the abundance and stability of recovered associated factors, comprehensive proteomic analyses using tagged subunits have indicated that individual Mediator subunits can display distinct interaction networks, suggesting functional specialization within the complex. For example, 42 proteins were identified as exclusively interacting with MED16 (Figure 3B) [23,91]. In particular, MED16 can dissociate from the core Mediator and form alternative transcriptional regulatory complex with upstream-binding protein 1 and transcription factor CP2, enabling context-dependent gene activation or repression that, however, still relied on intact Mediator complex [91]. This IP-MS approach has thereby revealed that Mediator complex, through MED23, participates in multiple regulatory processes, including alternative splicing [73], monoubiquitylation of histone H2B [75], and transcriptional termination [23]. In addition, the MED26 subunit has been linked to Pol II elongation through interactions with super-elongation complex subunits [22]. Proteomic analyses in *Saccharomyces cerevisiae* [92], *Caenorhabditis elegans* [93], *Tetrahymena thermophila* [94], and *Arabidopsis thaliana* [95] further revealed that purification of distinct Mediator subunits yields partially divergent interaction landscapes. Although most-copurifying proteins likely reflect canonical Mediator functions [96], quantitative differences suggest that subunits may occupy distinct molecular environments and imbalance of cellular abundance (Figure 3C). The assembly state of numerous protein complexes as well as their abundance dynamically change to respond functionally to specific environmental stimuli [97]. For example, MED21 is overall the most abundant protein component in the Middle module, which suggests that it is present at more than one copy per Mediator complex or exists outside of Mediator [98]. On the other hand, MED25 is present at substoichiometric levels and is easily dissociated from Mediator and MED26 is mutually exclusive with CKM–MED association (Figure 3D) [45,62,98,99]. Altered Mediator dynamics have also been observed during heat shock in yeast [60,100,101] and upon nutrient signals in mouse liver [102]. Such observations are consistent with a model in which non-canonical functions may arise from redistribution of a fraction of the subunit pools and/or alternative assembly states.

## Conclusion

The expanding functional landscape of Mediator complex reflects not only its central role in transcription but also its capacity to operate in distinct cellular contexts. While many subunit specific functions appear inseparable from the intact complex, some subunits, such as MED19 and MED12, appear to exhibit independent functionality outside it. Whether these examples are isolated phenomena or merely the first glimpses of a broader trend remains a compelling question for future investigation. These observations reframe multiprotein complexes not merely as integrated static molecular machines but as reservoirs of new regulatory potential. Within these complexes, architectural hierarchy, dynamic assembly, cellular localization, and quantitative heterogeneity enable functional diversification at the level of individual subunits. Key questions remain. Do non-canonical functions arise through regulated disassembly or from pre-existing pools of free subunits and how prevalent is subunit specialization within Mediator and other cellular machines?

## Perspectives

- **Importance in the field:** This review redefines our understanding of how multiprotein complexes generate functional diversity by highlighting the existence of subunit independent activity, using Mediator as a model system. These findings have broad implications for gene regulation and disease mechanisms.
- **Current thinking:** Subunits are generally viewed as integral components whose functions depend on the intact multiprotein complex and its overall architecture.
- **Future directions:** Mediator complex provides a powerful model system to investigate how widespread subunit specialization is and uncover mechanisms that lead to independent subunit activity. Do non-canonical functions drive disease?

## Open Access

Open access for this article is provided via our Subscribe to Open programme, made possible through the support of our subscribing institutions. Learn more here: https://portlandpress.com/pages/open_access_options_and_prices#conditional.

## Abbreviations

CDK8: Cyclin-dependent kinase 8
CKM: CDK8 kinase module
IP-MS: Immunoprecipitation-mass spectrometry
MED: Mediator
PIC: Pre-initiation complex
RNA Pol II: RNA polymerase II
TGF-βR2: Transforming growth factor beta receptor 2
